# Short-Term Outcomes in Patients Undergoing Paraesophageal Hiatal Hernia Repair

**DOI:** 10.1038/s41598-020-61566-2

**Published:** 2020-04-30

**Authors:** Raelina S. Howell, Helen H. Liu, Patrizio Petrone, María Fernanda Anduaga, María José Servide, Keneth Hall, Alexander Barkan, Shahidul Islam, Collin E. M. Brathwaite

**Affiliations:** 1 Department of Surgery, NYU Winthrop Hospital, Mineola, NY USA; 2 Department of Biostatistics, NYU Winthrop Hospital, Mineola, NY USA

**Keywords:** Oesophagus, Stomach, Nausea

## Abstract

Many patients with hiatal hernias (HH) are asymptomatic; however, symptoms may include heartburn, regurgitation, dysphagia, nausea, or vague epigastric pain depending on the hernia type and severity. The ideal technique and timing of repair remains controversial. This report describes short-term outcomes and readmissions of patients undergoing HH repair at our institution. All patients who underwent HH repair from January 2012 through April 2017 were reviewed. Patients undergoing concomitant bariatric surgery were excluded. 239 patients were identified and 128 were included. Eighty-eight were female (69%) and 40 were male (31%) with a mean age of 59 years (range 20–91 years) and a mean BMI of 29.2 kg/m^2^ (17–42). Worsening GERD was the most common presenting symptom in 79 (61.7%) patients. Eighty-four laparoscopic cases (65.6%) and 44 robotic assisted (34.4%) procedures were performed. Mesh was used in 59 operations (3 polytetrafluoroethylene; 56 biologic). All hiatal hernia types (I-IV) were collected. Majority were initial operations (89%). Techniques included: Toupet fundoplication in 68 cases (63.0%), Nissen fundoplication in 36 (33.3%), Dor fundoplication in 4 (3.7%), concomitant Collis gastroplasty in 4 (3.1%), and primary suture repair in 20 (15.6%). Outcomes between robotic and laparoscopic procedures were compared. Length of stay was reported as median and interquartile range for laparoscopic and robotic: 1.0 day (1.0–3.0) and 2.0 days (1.0–2.5); p = 0.483. Thirty-day readmission occurred in 9 patients, 7 (8.3%) laparoscopic and 2 (4.6%) robotic; p = 0.718. Two 30-day reoperations occurred, both laparoscopic; p = 0.545. Total of 16 complications occurred; 18.6% had a complication with the use of mesh compared to 8.7% without the use of mesh, p = 0.063. There were no conversion to open modality and no mortalities were reported. Hiatal hernia repair can be performed safely with a low incidence of complications.

## Introduction

The true prevalence of hiatal hernias (HH) is difficult to estimate due to the fact that many patients are asymptomatic and diagnostic criteria are variable^1^. However, HH may be a large contributor to the pathophysiology of gastroesophageal reflux disease (GERD) and have been reported in up to 94% of patients with reflux esophagitis compared to 59% in the general population^2^. Hiatal hernias have also been shown to be present in almost 40% of morbidly obese patients^3^. Many HH patients are asymptomatic; though, symptomatic presentations include heartburn, regurgitation, dysphagia, nausea, or vague epigastric pain depending on the hernia type and severity. Hiatal hernias are classified into four types (I-IV), with type I comprising up to 95% of all hernias. Type I, also known as a sliding hernia, occurs when the gastroesophageal junction (GEJ) herniates above the diaphragm. Type II occurs when part of the gastric fundus herniates above the hiatus alongside a fixed GEJ. A combination of types I and II results in a type III and type IV ensues when a solid organ herniates through the hiatas (e.g. colon, spleen). Types II-IV are termed “paraesophageal hernias” and can be associated with significant clinical complications, such as ulceration, bleeding, gastric volvulus, and gastric perforation^4,5^.

Surgical indications are based on the hernia type and severity of presentation. Although laparoscopic repair has become the standard due to its superior outcomes compared to open repair, the optimal technique and timing of repair remains controversial^6^. Currently, many surgeons employ either the laparoscopic or robot-assisted approach with comparable efficacious and safe results^7,8^. The addition of crural reinforcement has short-term advantages compared to non-reinforced repairs; however, the advantages fail to persist with long-term patient follow up, resulting in a lack of clear evidence to support the routine use of mesh^6,9^.

Most reports have focused on outcomes such as recurrence rates, patient satisfaction, and long-term quality of life assessments^10–14^. However, short-term outcomes have become important in all forms of surgery. In this era of pay-for-performance or prospective payment modeling, optimization of 30-day indicators, including readmissions, is paramount. The need to focus on quality measures to maximize patient care is essential. This report presents our experience.

## Methods

A prospectively-maintained patient database was retrospectively queried for all cases of isolated HH repair from January 2012 through April 2017 on the foregut surgery service at a single institution. Patients undergoing concomitant bariatric surgery were excluded. Information obtained from the database included demographics (e.g. gender, age, body mass index [BMI]), surgical details (e.g. repair modality, mesh use, repair technique), and 30-day outcomes (e.g. length of stay [LOS], readmissions, reoperations, complications). The rationale for the utilization of robotic versus laparoscopic surgery was determined by several factors, including the surgeon’s experience and comfort, patient preference, and achievement of the optimal outcome for the patient. The use of mesh was decided based on a combination of the following factors: long-standing hernia, large hernia size, revision surgery, and if the crural fibers appeared attenuated or weak at the time of surgery. In the early period of the study, synthetic mesh was preferred by some surgeons. Outside of surgeon preference, cost of product with or without insurance coverage was another factor. Of note, biologic mesh was not used in the setting of large size defects that required bridging for crural apposition.

Demographic and clinical characteristics were summarized by group using mean (standard deviation), median (interquartile range) or frequency (percentage) as appropriate. Continuous variables were assessed for normality using Kolmogorov-Smirnov test and visual plots, such as histograms and Q-Q plots. Continuous variables that followed normal distribution were compared using two-sample t-test, and non-normally distributed variables were compared using Wilcoxon rank-sum test. Fisher’s exact test was used to compare categorical variables. P-values less than 0.05 were considered statistically significant. SAS 9.4 was used to perform all analyses.

This study was approved by the NYU Winthrop Hospital Institutional Review Board (IRB) and all methods were carried out in accordance with relevant guidelines and regulations. Informed consent was not required by the IRB due to the retrospective, de-identified nature of the chart-review study.

## Results

Two hundred and thirty-nine patients were initially identified. Of these, 111 concomitant bariatric surgery cases were excluded and 128 patients were included in the final analysis. Table 1 shows the demographic summary statistics of the included patients.

**Table 1:** Demographics and Clinical Characteristics.

Variable	Laparoscopic (N = 84)	Robotic (N = 44)	P-value^†^
Gender, n(%)			1.000
Male	26(31)	14(31.8)	
Female	58(69)	30(68.2)	
Age (years), median(IQR)	61(50–70.5)	63(51.5–72.5)	0.391
BMI (kg/m^2^), mean ± SD	29.9 ± 5.8	28.1 ± 4.9	0.086
Surgery Fundoplication, n(%)			1.000
Fundoplication	71(84.5)	37(84.1)	
Suture Repair	13(15.5)	7(15.9)	
Fundoplication subtype, n(%)*			0.812
Toupet	46(64.8)	22(59.5)	
Nissen	22(31)	14(37.8)	
Dor	3(4.2)	1(2.7)	
Mesh Used, n(%)	35(41.7)	24(54.6)	0.193
Initial vs revision procedure, n(%)			0.042
Initial	79(94.1)	35(79.6)	
Revision	5(5.9)	9(20.4)	
Hernia Type, n(%)^‡^			1.000
I	3(3.8)	2(4.7)	
II	7(8.9)	4(9.3)	
III	68(86.0)	36(83.7)	
IV	1(1.3)	1(2.3)	
GERD, n(%)	50(61.7)	29(69.1)	0.552

^†^P-values are from two sample ttest for normally distributed continuous variables, Wilcoxon rank-sum test for non-normally distributed variables and Fisher’s exact test for categorical variables.

*Denominators for this subtype is 71 for laparoscopic and 37 for robotic.

^‡^Denominators for the hernia type is 79 for laparoscopic and 43 for robotic. Six were unknown due to inconclusive imaging and lack of specification in the operative report.

Patient outcomes between those undergoing laparoscopic and robotic procedures were compared (Table 2). Hiatal hernias were diagnosed preoperatively either radiographically (computed tomography or upper gastrointestinal series) or endoscopically (esophagogastroduodenoscopy). Of note, crural reinforcement with mesh was performed in approximately 50% of the repairs. Fifty-six hernias were reinforced with biologic mesh – 51 porcine urinary bladder matrix grafts, 4 bovine acellular dermal matrix grafts, and 1 fully absorbable poly-4-hydroxybutyrate composite mesh. Three hernias were reinforced with synthetic, polytetrafluroethylene grafts.

**Table 2:** Comparisons of outcomes between robotic and laparoscopic procedures.

Variable	Laparoscopic (N = 84)	Robotic (N = 44)	P-value*
Length of stay (day), median(IQR)	1.0 (1.0–3.0)	2.0(1.0–2.5)	0.483
30-day readmission, n(%)	7(8.3)	2(4.6)	0.718
30-day reoperation, n(%)	2(2.4)	0(0)	0.545
Complication, n(%)	10(11.9)	6(13.6)	0.784

*P-values are from Fisher’s exact test for categorical variables and Wilcoxon rank-sum test for continuous variables.

Neither modality was associated with 30-day readmission or reoperation. There were no mortalities noted. Additional analysis was performed looking at postoperative complications with the use of mesh or no mesh (Table 3). There were 18.6% (11/59) that had a complication with the use of mesh compared to 8.7% (5/69) among those without the use of mesh, p = 0.063.

**Table 3:** Postoperative complications (N = 16) categorized by mesh or no mesh use by Clavien-Dindo classification.

Clavien-Dindo Score	Mesh (N = 11)	No Mesh (N = 5)
I	5(45.4%)	2(40.0%)
II	1(9.1%)	2(40.0%)
IIIa	4(36.4%)	0(0.0%)
IIIb	1(9.1%)	1(20.0%)
IV	0(0.0%)	0(0.0%)
V	0(0.0%)	0(0.0%)

Complications were graded according to the Clavien-Dindo classification system^15^. Clavien-Dindo ranking is based on the therapy used to treat complications and are divided into five groups, I-V, ranging from therapeutic regimens in grade I (e.g. antiemetics) to death in grade V. Complications encountered in this study included: grade I (post-operative hypoxemia requiring new home oxygen, narcotic-induced lethargy, delayed gastric emptying resolved with bowel rest, two self-limiting, small pneumothoraces, and two failed trial of voids requiring foley reinsertion), grade II (anticoagulation for one new-onset atrial fibrillation, one deep vein thrombosis, and one aspiration pneumonia requiring intravenous antibiotics), grade IIIa (one thoracentesis for bilateral pleural effusions and three esophagoduodenoscopies for dysphagia), and grade IIIB (one re-operation for incarcerated ventral hernias and one re-operation for diaphragmatic bleeding).

## Discussion

Pay-for-performance (PFP) incentives have been implemented at numerous hospitals in recent years in an attempt to facilitate improved patient outcomes via provider performance. As such, a focus on short-term outcomes (e.g. 30-day outcomes, readmissions, complications) has become important. Several international studies have evaluated the effect of PFP on patient outcomes. In England, 1.8 million hospital admissions to 24 hospitals over a five-year period were assessed for reductions in 30-day mortality with and without the PFP model^16^. A significant reduction in mortality was seen in conditions that were both covered and not covered by the incentive program, highlighting a potential positive spillover effect of provider care. However, during long-term follow up, mortality reductions at the PFP hospitals were no longer significantly different than the non-PFP hospital rates. In Sweden, PFP was implemented for primary care and the effect on registry entry and data completeness was assessed^17^. PFP led to an increase in entries and data completion; however, confounding may have been present during comparison to pre-PFP data. A recent systematic review of the effects of PFP on processes of care, health, and healthcare use reported a possible association with improved processes of care in the ambulatory setting, with limited, inconsistent results for long-term effects or effects on health outcomes^18^. PFP was also analyzed for its effect specifically on surgical outcomes (serious complications and 30-day mortality) in 12 US states^19^. No significant improvement in surgical outcomes was found comparing PFP and non-PFP hospitals.

The advantage of mesh reinforcement of cruroplasty during HH repairs remains a controversial subject in the literature due to the lack of consensus definitions of outcomes such as recurrence and complication. Several recent meta-analyses and literature reviews have varying results, with some finding no difference in outcomes (reoperation and complication rates) comparing mesh to suture reinforcement^9,20,21^, but also finding a significant reduction in hernia recurrence with the use of mesh^20,22,23^. Furthermore, studies have also compared the efficacy of biologic versus synthetic mesh. Synthetic and biologic mesh has been associated with erosion, fibrosis, migration, esophageal stenosis, and stricture^24^. Csatelijns *et al*. performed a literature review from 2004 to 2015 and found a significantly lower recurrence rate and higher complication rate with the use of synthetic mesh^25^; while Zhang *et al*. reported that biologic mesh was associated with improved short-term quality of life and a higher incidence of dysphagia^23^. A majority of these studies agree that more long-term follow-up studies are needed with a continued focus on reducing symptomatic outcomes.

Readmissions have become a major metric by which the performance of hospitals are ranked as readmissions usually represent an adverse event for the patient. A recent study evaluated trends in postoperative 30-day readmissions over a 10-year period in the Veterans Health Administration (VA) population^26^. Nine surgical specialties were included: general, urology, neurosurgery, orthopedic, otolaryngology, plastic, thoracic, peripheral vascular, and cardiac. Readmission rates varied from 9% (urology) to 16.6% (cardiac) and the overall 30-day readmission rate was found to significantly decrease from 12.9% to 12.2% (p = 0.04) over the 10-year period. The most common causes for readmission were postoperative infection, urinary tract infection, and digestive system complications. Although the study did not include patients readmitted to non-VA hospitals, the study highlighted the importance of readmission rates in terms of quality of care and associated medical costs. Among Medicare beneficiaries in 2009, 30-day readmission rates were found to be 21% after discharge for a medical condition and 15.6% after surgical procedures^27^. Subsequently, a 2013 study reviewed 30-day readmissions delineated by surgical subspecialty using the National Surgical Quality Improvement Program data in an attempt to develop a predictive model for readmission^28^. Overall readmission was 7.8%, ranging from 5% (general surgery) to 15.8% (hepatobiliary). The authors suggest that scoring based on American Society of Anesthesiologists class and LOS may assist in risk stratification and early patient intervention.

While overall our findings indicate no increased risk of 30-day readmission or complications associated with minimally-invasive HH repair, further analysis demonstrates that the use of mesh trends towards increased rates of surgical complications over no mesh (18.6% versus 8.7%). If this trend is born out in the direction of increased complications with the use of mesh, it highlights the importance for the Centers for Medicare and Medicaid Services (CMS) payers to look closer at potential short-term complications besides hernia recurrence. A recent study by Hall *et al*. focused on patient-centered quality of life post-laparoscopic HH repair and report improvement in reflux symptoms, but worsening postoperative dysphagia scores within the first 6 weeks, mirroring that of preoperative values^29^. While the use of mesh is largely accepted, those that require HH repair often carry multiple comorbidities placing them at poorer functional status and increased risk for 30-day morbidity^30^. In light of the focus on readmission for reimbursement by the CMS, further studies are needed to implement strategies for risk reduction and improved patient outcomes, thereby lowering 30-day readmissions. Limitations of our study include the small number of patients and the inability to perform cohort matching.

Many studies have presented supportive short-term results comparing the use of mesh to non-mesh HH repair. However, mixed hernia recurrence rates have been reported over a longer follow-up period, calling into question the benefit of mesh repair^9–14^. CMS value-based programs have been touted as quality financial incentives to reduce readmissions and model pay-for-performance. Our results indicate no increased risk of 30-day readmission or complications associated with minimally invasive HH repair, but highlight the need for future studies to observe the impact of mesh on short-term complications in order to continue to improve structural metrics of hospital and surgical quality^21^.

## Conclusion

Minimally-invasive hiatal hernia repair is safe and feasible. The use of mesh-reinforced crural repair does not adversely affect short-term outcomes such as 30-day readmission but may trend towards an increased rate of short-term complications. The focus of this manuscript was short-term outcomes; however, long-term follow up is essential to delineate any future significant impact of method or modality of repair on patient outcome. Future, prospective studies are needed to obtain high-quality, standardized data.
